# Mass Screening of SARS-CoV-2 Variants using Sanger Sequencing Strategy in Hiroshima, Japan

**DOI:** 10.1038/s41598-022-04952-2

**Published:** 2022-02-14

**Authors:** Ko Ko, Kazuaki Takahashi, Shintaro Nagashima, Bunthen E, Serge Ouoba, Md Razeen Ashraf Hussain, Tomoyuki Akita, Aya Sugiyama, Takemasa Sakaguchi, Hidetoshi Tahara, Hiroki Ohge, Hideki Ohdan, Tatsuhiko Kubo, Nobuhisa Ishikawa, Toshiro Takafuta, Yoshiki Fujii, Michi Mimori, Fumie Okada, Eisaku Kishita, Kunie Ariyoshi, Masao Kuwabara, Junko Tanaka

**Affiliations:** 1 Department of Epidemiology, Infectious Disease Control and Prevention, Graduate School of Biomedical and Health Science, Hiroshima University, 1-2-3, Kasumi, Minami-ku, Hiroshima, 734-8551 Japan; 2 Payment Certification Agency, Ministry of Health, Phnom Penh, Cambodia; 3 Unité de Recherche Clinique de Nanoro (URCN), Institut de Recherche en Science de la Santé (IRSS), Nanoro, Burkina Faso; 4 Department of Virology, Graduate School of Biomedical and Health Sciences, Hiroshima University, Hiroshima, Japan; 5 Department of Cellular and Molecular Biology, Graduate School of Biomedical and Health Sciences, Hiroshima University, Hiroshima, Japan; 6 Department of Infectious Diseases, Hiroshima University Hospital, Hiroshima, Japan; 7 Department of Gastroenterological and Transplant Surgery, Graduate School of Biomedical and Health Sciences, Hiroshima University, Hiroshima, Japan; 8 Department of Public Health and Health Policy, Graduate School of Biomedical and Health Sciences, Hiroshima University, Hiroshima, Japan; 9 Department of Respiratory Medicine, Hiroshima Prefectural Hospital, Hiroshima, Japan; 10 Hiroshima City Funairi Citizens Hospital, Hiroshima, Japan; 11 Hiroshima City Institute of Public Health, Hiroshima, Japan; 12 Hiroshima City Health and Welfare Bureau, Hiroshima, Japan; 13 Hiroshima Prefectural Health and Welfare Bureau, Hiroshima, Japan; 14 Hirishima Prefectural Technology Research Institute Health and Environment Center, Hiroshima, Japan; 15 Hiroshima Prefectural Center for Disease Control and Prevention, Hiroshima, Japan

**Keywords:** Epidemiology, PCR-based techniques

## Abstract

This study aimed to develop the feasible and effective universal screening strategy of the notable SARS-CoV-2 variants by Sanger Sequencing Strategy and then practically applied it for mass screening in Hiroshima, Japan. A total of 734 samples from COVID-19 confirmed cases in Hiroshima were screened for the notable SARS-CoV-2 variants (B.1.1.7, B.1.351, P.1, B.1.617.2, B.1.617.1, C.37, B.1.1.529, etc.). The targeted spike region is amplified by nested RT-PCR using in-house designed primer set hCoV-Spike-A and standard amplification protocol. Additionally, randomly selected 96 samples were also amplified using primer sets hCoV-Spike-B and hCoV-Spike-C. The negative amplified samples were repeated for second attempt of amplification by volume-up protocol. Thereafter, the amplified products were assigned for Sanger sequencing using corresponding primers. The positive amplification rate of primer set hCoV-Spike-A, hCoV-Spike-B and hCoV-Spike-C were 87.3%, 83.3% and 93.8% respectively for standard protocol and increased to 99.6%, 95.8% and 96.9% after second attempt by volume-up protocol. The readiness of genome sequences was 96.9%, 100% and 100% respectively. Among 48 mutant isolates, 26 were B.1.1.7 (Alpha), 7 were E484K single mutation and the rest were other types of mutation. Moreover, 5 cluster cases with single mutation at N501S were firstly reported in Hiroshima. This study indicates the reliability and effectiveness of Sanger sequencing to screen large number of samples for the notable SARS-CoV-2 variants. Compared to the Next Generation Sequencing (NGS), our method introduces the feasible, universally applicable, and practically useful tool for identification of the emerging variants with less expensive and time consuming especially in those countries where the NGS is not practically available. Our method allows not only to identify the pre-existing variants but also to examine other rare type of mutation or newly emerged variants and is crucial for prevention and control of pandemic.

## Introduction

Coronavirus disease 2019 (COVID-19), the respiratory illness responsible for the COVID-19 pandemic, is caused by severe acute respiratory syndrome coronavirus 2 (SARS-CoV-2)^1^. SARS-CoV-2 is a positive sense single stranded RNA virus and is believed to be the animal origin as its genetic structure is closely related to bat coronavirus^1,2^. Since the first confirmed cluster of cases of COVID-19 in the late-December 2019 in Wuhan, China^3,4^, total 261 million cases had been reported worldwide as of November 29, 2021. This pandemic took away the 5.2 million lives and the new cases are still increasing at 555 K cases per 7 days^5^.

Over a year outbreak, various types of SARS-CoV-2 variants emerged days by days and some variants are dominant over the wild type causing burst outbreaks in particular area. The variants were identified by Phylogenetic Assignment of Named Global Outbreak Lineages (PANGO Lineage)^6^ as described in the Global Initiative on Sharing All Influenza Data (GISAID: https://www.gisaid.org). Three notable SARS-CoV-2 variants were appeared since November 2020, sharing the same mutation of N501Y and are called B.1.1.7 (VOC Alpha GRY/501Y.V1), B1.351 (VOC Beta GH/501Y.V2) and P.1 (VOC Gamma GR/501Y.V3) which had high ACE2 binding affinity^7^. The change in transmission pattern, duration, the peak and the severity of disease were coincidently observed along with the emergence of 501Y variants^8^. Later, the B.1.429 (VOI Epsilon GH/452R.V1), B.1.617.2, AY.1 and AY.2 (VOC Delta G/478 K.V1) and B.1.617.1 (VOI Kappa G/452R.V3), B.1.525 (VOI Eta G/484 K.V3), P.3 (VOI Theta GR/1092 K.V1P.3) and C.37 (VOI Lambda GR/452Q.V1) were continuously emerged. Additionally, the Delta plus variant was also notified in India. These all mutations were in spike region of the virus which serves as the initial checkpoint to enter the host cell through binding with specific receptor and fusion^9^. On November 26, 2021, WHO notified the emergence the new SARS-CoV-2 variant called B.1.1.529 (Omicron) variant. Therefore, understanding the molecular characterization and its mutation pattern are critically important to set up the effective strategies on prevention and control.

Although the Next Generation Sequencing (NGS) strategies are widely used for identification of SARS-CoV-2 virus by reconstruction of nearly or complete full-length genomes^10^ which can be analyzed for viral evolution and quasispecies^11^, it requires the advanced technologies, skillful human resources, expensive and time consuming so that approximately 2% of SARS-CoV-2 strains have been reported in GISAID. Considering the low reporting rate of viral genome for identification of emerging variants, our study aimed to develop the feasible and effective universal screening strategy of the notable SARS-CoV-2 variants by Sanger sequencing and practically applied it for mass screening in Hiroshima, Japan.

## Methods

### Subjects of the study

**Figure 1:**
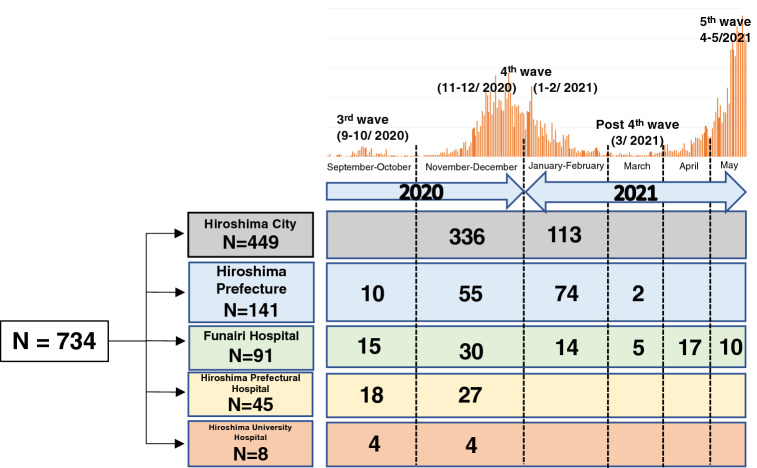
Flow of study subjects in Hiroshima, Japan. The figure showed the sources of the study subjects included in this study during each wave outbreak of SARS-CoV-2 in Hiroshima.

Total 734 samples (287 nasopharyngeal swab and 447 saliva) from the confirmed cases of COVID-19 collected from different cities of Hiroshima prefecture during September 1, 2020 to May 25, 2021 were included in this study. The flow of study subjects was fully explained in Fig. 1 and a part of samples were provided from Hiroshima City Institute of Public Health. The rest were collected from three hospitals: Hiroshima University Hospital, Funairi Hospital and Hiroshima Prefectural Hospital, all which were included in five main COVID-19 treatment centers in Hiroshima Prefecture.

### Nucleic acid extraction and quantitative measurement of SARS-CoV-2

50 μL of sample from COVID-19 confirmed cases were subjected to nucleic acid extraction using SMITEST EX-R&D (MBL, USA) and the final pellet were dissolved in 50 μL of RNase free water. Then, 10% (5 μL) of the extracted template RNA was used to measure the viral titer quantitatively using nucleocapsid (N) protein specific primers NIID_2019-n-CoV-N-F2 (nt29, 125-nt29, 144) and NIID_2019-nCoV-N-R2 (nt29, 999-nt29, 280) and the probe NIID_2019-nCoV-N-P2 (nt29, 222-nt29, 241) and the standard template of known concentration at serial dilution from 10^8^ to 1 copy and the negative control by mean of the real-time reverse transcriptase polymerase chain reaction (qRT-PCR)^12^. The measured values of viral titer were transformed into number of copies per milliliter.

### Standard protocol for the amplification of spike region by nested RT-PCR

5% (2.5 μL) of template RNA was used to amplify the spike protein of SARS-CoV-2 using the appropriate in-house developed primer sets by mean of nested reverse transcriptase polymerase chain reaction (RT-PCR). The first round of nested RT-PCR was done by Prime Script One-Step RT- PCR kit Ver.2 (Takara Bio Inc., Shiga, Japan) and the thermal cycle was as follows: reverse transcription at 50 °C for 30 min and pre-denaturation at 94 °C for 1 min, denaturation at 95 °C for 30 s, annealing at 55 °C for 30 s, and extension at 72 °C for 1 min with 40 cycles, the final extension was 7 min at 72 °C. Then, the second round of nested RT-PCR was done by TaKaRa Ex Taq Hot Start version (Takara Bio Inc., Shiga, Japan) using the 20% (5 μL) of first round nested RT-PCR product and the thermal cycle was as follows: denaturation at 98 °C for 10 s, annealing at 55 °C for 30 s, and extension at 72 °C for 1 min with 30 cycles, the final extension was 7 min at 72 °C. The amplified nested RT-PCR product was examined by gel electrophoresis using 3% of 1:3 Agarose Gel and running at 150 V for 30 min.

### Volume-up protocol for the amplification of previously negative samples by standard protocol

If the nested RT-PCR by first attempt was negative, the samples were reassigned for nucleic acid extraction from 50 μL of original samples as per aforementioned SMI-TEST. The final pellet was dissolved in the reagent mixture containing 2 μL mixture of forward and reverse primer (10 pmol/μL), 12.5 μL PrimeScript 1 step buffer, 1 μL PrimeScript 1 step enzyme, 9.5 μL distilled H_2_O and underwent the first round nested RT-PCR of same thermal cycles so that 100% of the extracted template RNA was attempted for volume up reaction. Then, the second round nested RT-PCR was done as per the abovementioned method.

### In-house developed primer sets for variant screening by Sanger sequencing strategy

**Figure 2:**
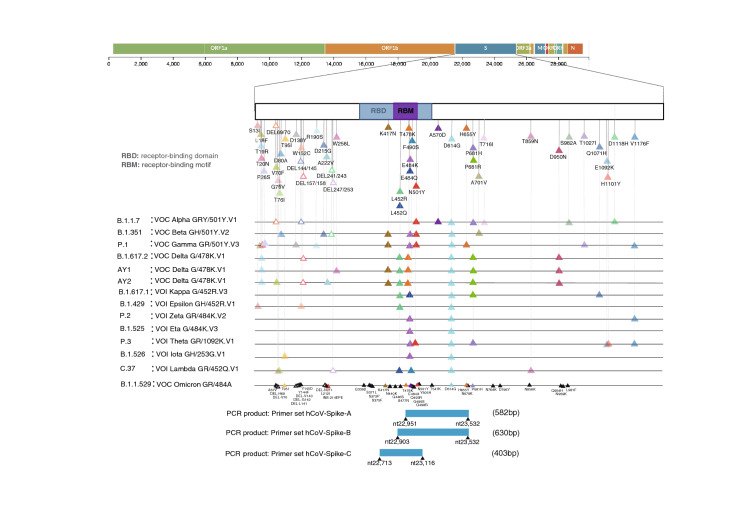
Schematic mutation pattern of notable SARS-COV-2 variants and its primer product. This figure explained the classification criteria for the identification and screening of B.1.1.7 (Alpha), B.1.351 (Beta), P.1 (Gamma), B1.617.2 (Delta), B.1.617.1 (Kappa), C.37 (Lambda and B.1.1.529 (Omicron). All those mutations were in the spike region and the targeted fragment of SARS-CoV-2 was amplified by primer set hCoV-Spike A, hCoV-Spike-B and hCoV-Spike-C so that the expected genome sequences were also shown.

1. **Primer set hCoV-Spike-A for screening of 501Y related variants (B.1.1.7 (Alpha), B.1.351 (Beta) and P.1 (Gamma) variants)**
  The primer set hCoV-Spike-A was fully described in Table 1a and it covers the spike region from nt22951 to nt23532. This primer set is used to identify Alpha, Beta or Gamma using the classification checkpoints as shown in Fig. 2. All 734 samples from Hiroshima underwent amplification using primer set hCoV-Spike-A.
2. **Primer set hCoV-Spike-B for screening of B.1.429 (Epsilon), B.1.617.2 (Delta), B.1.617.1 (Kappa), C.37 (Lambda) and newly emerged B.1.1.529 (Omicron)**
  The primer set hCoV-Spike-B covers the spike region from nt22903 to nt23532 (Table 1a). This primer set is used to identify B.1.429 (Epsilon), B.1.617.2 (Delta), B.1.617.1 (Kappa), C.37 (Lambda) and newly emerged B.1.1.529 (Omicron) using the classification checkpoints as shown in Fig. 2. Randomly selected 96 samples were used to examine the function of primer set hCoV-Spike-B.
3. **Primer set hCoV-Spike-C for subclassification of B.1.617.2 (Delta) and AY.1, AY.2 (Delta plus)**
  The primer set hCoV-Spike-C is designed to translate the spike region from nt22713 to nt23115 (Table 1a).

**Table 1:** SARS-CoV-2 specific primers used for partial sequences of particular region.

	Stage polarity	Primer name	Nucleotide position	Nucleotide sequence (5′-3′)	
(a) Primers used in polymerase chain reaction					
hCoV-Spike-A (Ver.1)	PCR 1st Sense	SP31S	22,882–22,902	TCTTGATTCTAAGGTTGGTGG	
	PCR 1st Sense	SP32S	22,904–22,927	AATTATAATTACCTGTATAGATTG	
	PCR 1st Antisense	SP35AS	23,612–23,631	TGACTAGCTACACTACGTGC	
	PCR 1st Antisense	SP36AS	23,577–23,598	TTAGTCTGAGTCTGATAACTAG	
	PCR 2nd Sense	SP07S	22,929–22,950	TTTAGGAAGTCTAATCTCAAACC	
	PCR 2nd Sense	SP33S	22,923–22,945	GATTGTTTAGGAAGTCTAATCTC	
	PCR 2nd Antisense	SP37AS	23,556–23,575	GCATATACCTGCACCAATGG	
	PCR 2nd Antisense	SP38AS	23,533–23,554	TATGTCACACTCATATGAGTTG	
hCoV-Spike-B (Ver.2)	PCR 1st Sense	SP45S	22,790–22,809	ATCGCTCCAGGGCAAACTGG	
	PCR 1st Sense	SP46S	22,834–22,855	ATTACCAGATGATTTTACAGGC	
	PCR 1st Antisense	SP35AS	23,612–23,631	TGACTAGCTACACTACGTGC	
	PCR 1st Antisense	SP36AS	23,577–23,598	TTAGTCTGAGTCTGATAACTAG	
	PCR 2nd Sense	SP31S	22,882–22,902	TCTTGATTCTAAGGTTGGTGG	
	PCR 2nd Sense	SP47S	22,858–22,879	CGTTATAGCTTGGAATTCTAAC	
	PCR 2nd Antisense	SP37AS	23,556–23,575	GCATATACCTGCACCAATGG	
	PCR 2nd Antisense	SP38AS	23,533–23,554	TATGTCACACTCATATGAGTTG	
hCoV-Spike-C	PCR 1st Sense	22632S	22,632–22,652	GAATCAGCAACTGTGTTGCTG	
	PCR 1st Sense	22659S	22,659–22,680	CTGTCCTATATAATTCCGCATC	
	PCR 1st Antisense	23170AS	23,170–23,193	TTGAAGTTGAAATTGACACATTTG	
	PCR 1st Antisense	23201AS	23,201–23,221	AGTAAGAACACCTGTGCCTG	
	PCR 2nd Sense	22687S	22,687–22,708	CACTTTTAAGTGTTATGGAGTG	
	PCR 2nd Sense	22712S	22,712–22,734	CCTACTAAATTAAATGATCTCTG	
	PCR 2nd Antisense	23117AS	23,117–23,138	CACAAACAGTTGCTGGTGCATG	
	PCR 2nd Antisense	23141AS	23,141–23,164	AACCAAATTAGTAGACTTTTTAGG	

	Primer name	Nucleotide position	Nucleotide sequence (5′-3′)	Estimated length of PCR product	Target nucleotide position
(b) Primers used in region-specific partial sequencing					
hCoV-Spike-A (Ver.1)	SP38AS		TATGTCACACTCATATGAGTTG	582	22,951–23,532
hCoV-Spike-B (Ver.2)	SP38AS		TATGTCACACTCATATGAGTTG	630	22,903–23,532
hCoV-Spike-C (Ver.2)	22712S		CCTACTAAATTAAATGATCTCTG	403	22,713–23,116

This primer set is used to subclassify between the original Delta (B.1.617.2) and Delta plus (AY.1, AY.2) in Fig. 2. Randomly selected 96 samples were used to examine the function of primer set hCoV-Spike-C.

### Sanger Sequencing of SARS-CoV-2 spike protein partial genomes

The positive nested RT-PCR products were undergone Sanger Sequencing for partial genomes of targeted spike region with 3730xl DNA sequencer and BigDye Terminator v3.1 Cycle Sequencing Kit (Applied Biosystems, Foster City, CA, USA) and the corresponding primer set as shown in Table 1b.

### Validation of Sanger Sequencing Strategy for SARS-CoV-2 variant screening

To validate the absence of amplification induced mutation during 70 amplification cycles, we performed the amplification of previously submitted eight SARS-CoV-2 isolates from Hiroshima which had been done by the NGS and submitted at GISAID (accession number: EPI_ISL_855345 to EPI_ISL_855352 at GISAID) using the standard protocol for the nested RT-PCR and in-house developed primer sets. Each analysis was performed in quadruplicate (total 32 tests). We found 100% identity between the references and the new amplified products by Sanger sequencing. It validates not only the absence of amplification induced mutations but also agreement between Sanger sequencing and the NGS (results were not shown).

### Classification of the notable SARS-CoV-2 variants

The classification criteria were set to screen the notable SARS-CoV-2 variants as shown in Fig. 2.

The initial checkpoint was set at nucleotide position nt23063 and if we found mutation from adenine (A) to thymine (T) at nt23063, further identification was done as follows: double mutation of A23063T (referred to N501Y) and C23271A (referred to A570D) for B.1.1.7 (Alpha), G23013A (referred to E484K) and A23063T for B.1.351 (Beta) and triple mutation of G23012A, A23063T and C23525T (referred to H655Y) for P.1 (Gamma).

The secondary checkpoint was set at nucleotide position nt23012 when no mutation was found at primary checkpoint. If we found the mutation from Guanine (G) to Adenine (A) or Cystine (C)at nt23012, further identification was done as follows: double mutation of T22917G (referred to L452R) and C22995A (referred to T478K) for B.1.617.2 (Delta), T22917G (referred to L452R) and G23012C (referred to E484Q) for B.1.617.1 (Kappa) and T22917A (referred to L452Q) and T23031C (referred to F490S) for C.37 (Lambda).

The last checkpoint was set at nucleotide position nt22917 and if we found single mutation from Thymine (T) to Guanine (G) at nt22917 (referred to L452R) only, it is supposed to be B.1.429 (Epsilon).

When we found B.1.617.2 (Delta), further classification between the ordinary B.1.617.2 (Delta) and AY.1 or AY.2 (Delta Plus) can be done by checking at nt22813. The Delta variant without mutation at nt22813 is the ordinary B.1.617.2 (Delta) but it is AY.1 or AY.2 (Delta Plus) if there is mutation from Guanine (G) to Thymine (T) at nt22813 (referred to K417N).

The strain was defined as newly emerged B.1.1.529 (Omicron) variant if the various mutations were found as shown in Fig. 2 literally having K417N, T478K, E484A and N501Y mutation in the Sanger sequences.

### Genomic and statistical analysis

The raw genomes data were visually examined for the waveform using the ATGC-MAC and then the clean data was exported as FASTA files. The alignment was done using GENETYX-MAC ver. 21 (GENETYX COPORATION, Tokyo, Japan) and the mutation was checked in contrast to the reference SARS-CoV-2 genome (MN908947) retrieved from GenBank. The database was constructed, and the prevalence of notable SARS-CoV-2 variants were computed in Microsoft Excel.

### Ethic consideration

This study was approved by the Ethics Committee of Hiroshima University (E2122 and E2124). All participants provided the written informed consent at the beginning of the study. All procedures strictly adhered to the guidelines and the Declaration of Helsinki.

## Results

Total 734 samples collected from COVID-19 confirmed cases in Hiroshima Prefecture during September 1, 2020, through May 15, 2021, were included in this study. The viral titers of the samples ranged from 1 × 10^1^ to 4.21 × 10^8^ copies/mL.

### Positive rate of nested RT-PCR

**Figure 3:**
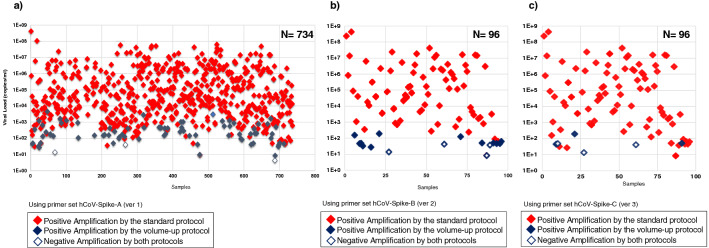
Viral load dependent postivity of nested PCR for spike region using **a)** primer set version 1, **b)** primer set version 2 and **c)** primer set version 3. The first scattered plot in **a)** showed the positive amplification versus the viral load for the primer set hCoV-Spike-A. The red colored dots represent those positive amplification by the standard protocol, the blue color for positive amplification by the volume-up protocol and the while color with blue marginated dots represent for the negative amplification by both protocols. The scattered plot in **b)** and **c)** showed for the primer set hCoV-Spike-B and hCoV-Spike-C respectively.

**Figure 4:**
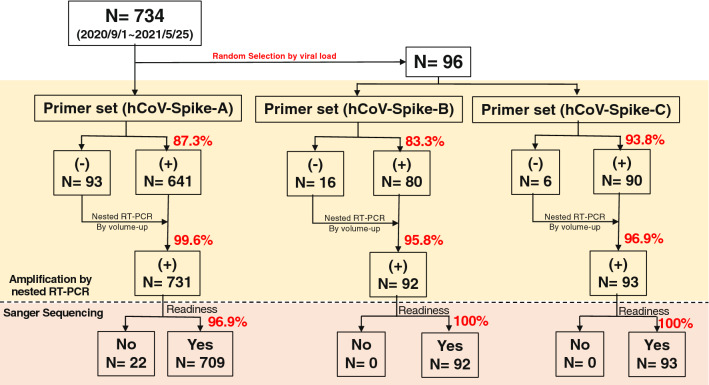
Amplification positive rate and readiness of Sanger sequences using primer set hCoV-Spike-A, hCoV-Spike-B and hCoV-Spike-C. This figure showed the positive rate of amplification by nested RT-PCR using the specific primer set hCoV-Spike-A, hCoV-Spike-B and hCoV-Spike-C respectively and also their readiness of partial genomes after direct sequencing.

All samples undergoing the RT-PCR using the primer set hCoV-Spike-A showed that 93 out of total 734 samples were negative amplification by the standard protocol so that the positive rate of nested RT-PCR was 87.3% (Figs. 3, 4). Most of those negative nested RT-PCR samples had low viral titer below 10^3^ copies/mL. After second attempt of nested RT-PCR by volume-up protocol, only three were negative amplification so that the positive amplification rate increased to 99.6% after volume-up reaction. Similarly, the positive amplification rate using primer set hCoV-Spike-B by standard protocol was 83.3% (16/96 were negative) and increased to 95.8% after volume-up reaction. Moreover, the positive amplification rate using primer set hCoV-Spike-C in the standard protocol was 93.8% (6/96 were negative) and increased to 96.9% after volume-up reaction. (Fig. 4).

### Readiness of targeted partial genomes among positive amplified products

Among 730 nested RT-PCR positive samples undergoing the Sanger Sequencing using primer set hCoV-Spike-A, 23 isolates were not able to analyze data because of many unidentified “N” in the sequences. Therefore, the readiness of the sequences using primer set hCoV-Spike-A was 96.9% whilst both the respective 92 and 93 amplified products using primer set hCoV-Spike-B and hCoV-Spike-C provided 100% readiness by Sanger Sequencing (Fig. 4).

### Distribution of the notable SARS-CoV-2 variants through the screening

**Figure 5:**
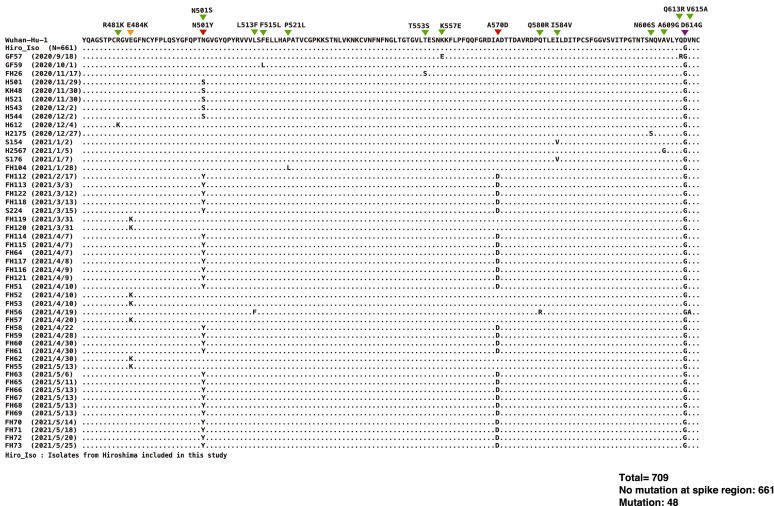
The mutation pattern found in selective screeing of the notable SARS-CoV-2 variants in Hiroshima. The figure showed the amino acid sequences translated from the genome sequences of the target spike region. The mutation points were showed with triangle on the uppermost row and all the genome sequences were compared to the reference strain (Wuhan-Hu-1) retrieved from GeneBank. All isolates from this study without any mutation at target fragment (N = 704) were shown as Hiro_Iso and those with mutation were shown individually.

**Figure 6:**
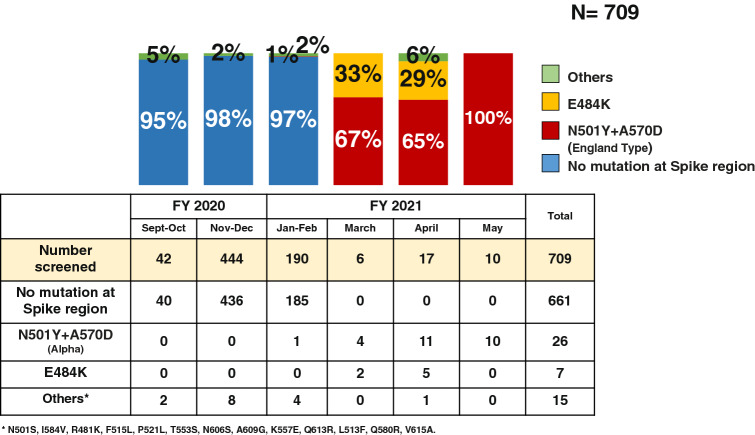
Prevalence of the notable SARS-CoV-2 variants in Hiroshima. The bar graph described the prevalence of B.1.1.7 (Alpha), E484K and the other forms of mutation found in Hiroshima Japan. The red color represents B.1.1.7 (Alpha), the orange color shows E484K mutation, the green color for the other forms of mutation and the blue color for those having no mutation at targeted spike region. The other forms of mutation include single mutations of N501S, I584V, R481K, F515L, P521L, T553S, N606S, A609G, double mutation of K557E and Q613R, and triple mutation of L513F, Q580R and V615A.

Among 709 isolates to be screened, 48 isolates had particular mutation as shown in Figs. 5 and 6 so that the overall mutation rate was 6.8%. SARS-CoV-2 variants were found in 5%, 2% and 3% respectively in September–October, November–December 2020, and January–February 2021 period. In March 2021, 67% were B.1.1.7 (Alpha) and the rest were single E484K mutation. In April 2021, 65%, 29% and 6% were B.1.1.7 (Alpha), E484K mutations and other forms of mutation. In May 2021, all of 10 isolates were found to be B.1.1.7 (Alpha). The other forms of mutation include single mutation at N501S, I584V, R481K, F515L, P521L, T553S, N606S, A609G, double mutation at K557E and Q613R, and the triple mutation at L513F, Q580R and V615A (Fig. 5).

## Discussion

Hiroshima, located in the southwest of Japan’s mainland and has a population of 2.8 million peoples^13^, has reported total 8,176 confirmed cases of COVID-19 with 115 attributable deaths as of May 15, 2021^14^. Five different waves of outbreaks were occurred within a year and the first three waves had maximum 26 cases per day with not more than 3 weeks duration for each outbreak and 1 month in-between gap. After mid-November 2020, the tremendous outbreak was occurred with the maximum of 141 confirmed cases per day. Hiroshima prefectural center for disease control reported that total 70 mutant isolates of SARS-CoV-2 since mid-February 2021. Among total, 43 isolates were B.1.1.7 (Alpha), and the remaining were still ongoing for the detail molecular analysis^12,14^.

Our study included the saliva samples which have reported sensitivity of 83.2% (95% CI 77.4–91.4%) and a pooled specificity of 99.2% (95% CI 98.2–99.8%) for the diagnosis of SARS-CoV-2^15^. The primer set hCoV-Spike-A used in this study was very convinced in amplification of targeted genomic fragment and the amplification rate was as high as 87.3% for the standard protocol and 99.6% for the second attempt of amplification by volume-up method. The standard protocol yielded negative amplification result only if the original viral titer was too low below 10^3^ copies/mL and it was fully covered by the modified volume-up reaction. Meanwhile, another primer set hCoV-Spike-B and hCoV-Spike-C also provided the high amplification rate as 83.3% and 93.8% for the standard protocol and 95.8% and 96.9% respectively after second attempt of amplification by volume-up method. The readiness of genomes was also high; 96.9% for primer set hCoV-Spike-A and 100% for both primers set hCoV-Spike-B and hCoV-Spike-C. These results proved for the effectiveness of the newly developed Sanger Sequencing Strategy and suggested that it is applicable universally for mass screening of SARS-CoV-2 variants.

Nowadays, the next generation sequencing (NGS) is used worldwide to reconstruct the full-length genomes, and to investigate the transmission chain and its origin, evolution, and emerging variants^16–18^. In GISAID, total 5,366,615 SARS-CoV-2 isolates were reported despite there are total 256,480,022 confirmed cases worldwide^5^. Therefore, full length genome sequences of SARS-CoV-2 can be performed by the NGS and reported only in 2% of all confirmed cases. As the whole Japan, 50,977 full length genome sequences of SARS-CoV-2 were reported in GISAID in spite of total 799,801 confirmed cases so that only 6.4% of cases were able to be examined for their full genomes. Therefore, it is clearly indicated that the NGS cannot be used to screen all samples for the emerging SARS-CoV-2 variants as the NGS is limited to those samples having low viral load (Ct > 25)^19^. Our method yielded more than 90% of both positive amplification rate and the readiness for all primer sets and can be applied in those samples with low viral load (< 10^3^ copies/mL) using the alternative volume-up method. In term of technology, cost and time taken for analysis, the Sanger Sequencing Strategy is feasible, less expensive and can provide the result in shorter duration compared to the NGS.

Moreover, the Ministry of Health, Labour and Welfare of Japan uses the real time RT-PCR based screening for N501Y and L452R mutation, such screening cannot identify the other types of mutant variants in contrast to our method. After one and half years of pandemic, the various types of mutations were reported with its clinical or virological significance so that the qRT-PCR based screening alone is not adequate to identify all SARS-CoV-2 variants. The Sanger Sequencing Strategy is based on the partial genomes sequencing of spike region enriched of the functional genomic information. Our study focused on the mutation in particular fragment of spike region as the spike of SARS-CoV-2 is the crucial point having the receptor binding domain protein and it is the initial landmark for viral binding, fusion and viral entry to the host cell^9,20,21^. Therefore, it serves as the immune recognition by host cell to initiate the cell mediated immune response. The basic idea of the development of vaccine and other immune mediated therapeutic agents is also emphasized on the spike region of SARS-CoV-2 virus. Therefore, it is essential to investigate the spike region mutation with any functional deterioration, impact on the vaccine development and its efficacy and also on the therapeutic agents. Sequencing less than 1kbp can provide the useful genomic information and high amplification rate so that it can be applied for the mass screening. Our study also provides the fundamental of universal primers for the partial sequencing of SARS-CoV-2 spike region.

On November 26, 2021, WHO announced the emergence of B.1.1.529 variant named Omicron having 31 mutations, 6 deletion and 1 insertion in the spike region. The Omicron variant possess the distinct mutation pattern having both K417N (Delta) and N501Y (Alpha) in the spike region plus E484A and various mutations which can be easily identified by our Sanger Sequencing Strategy (as shown in Fig. 2). Until now, the new SARS-CoV-2 variant are reported continuously, the variant screening is crucially required as the universal approach. Considering the countries where the standard NGS is not available either due to technical/ human resources insufficiency or cost effectiveness, the Sanger Sequencing Strategy introduced in this study has advantages over the NGS from all aspects. It is useful tool to notify the occurrence of new mutation or emergence of SARS-CoV-2 variant as well.

Our study provides the distribution pattern of SARS-CoV-2 variants in Hiroshima during the study period. SARS-CoV-2 having no mutation in the targeted spike region is circulated in Hiroshima until February 2021, after which E484K mutated strains and B.1.1.7 variant became dominant over previously reported strain. Then, B.1.1.7 variant became the main causative for the huge outbreak in the later May 2021. In addition to the notable SARS-CoV-2 variants, our study identified the different mutation pattern at nt23064 from Adenine (A) to Guanine (G) resulting in amino acid changes from Asparagine (N) to Serine (S) at aa501. No other report on this mutation pattern was found in GISAID until now and this mutation pattern was firstly notified in the sample collected on November 29, 2021 and the same mutation pattern was found in another 4 samples collected until December 2, 2020 (Fig. 4). The samples collection dates were very closed to one another, and it is believed that the cluster cases were occurred by this N501S mutant variant in Hiroshima during late November to early December 2020. But this mutation pattern was shortly disappeared and no longer detected in the later samples which indicated that this mutation type had weaker replication power, less virulence and transmissibility than other notable SARS-CoV-2 mutant variants.

Our study had some limitations. The study focused on the identification of notable SARS-CoV-2 variants so that the rare form of mutation in other regions can be missed. As the study used the Sanger Sequencing Strategy for getting partial genomes, the origin and the homology of the mutant variants found in this study cannot be ruled out. The further detailed molecular study on full genomes is required to find out those origin and homology. Moreover, understanding the genomic sequences and its mutation provides the scientific evidence and great input to the strategic planning and protocol change for the prevention, control and the effective countermeasure against COVID-19.

In conclusion, this study indicates the reliability and effectiveness of Sanger sequencing to screen large number of samples for the notable SARS-CoV-2 variants. Compared to the Next Generation Sequencing (NGS), our method introduces the feasible, universally applicable, and practically useful tool for identification of the emerging variants with less expensive and time consuming especially in those countries where the NGS is not practically available. Our method allows not only to identify the pre-existing variants but also to examine other rare type of mutation or newly emerged variants and is crucial for prevention and control of pandemic.

## Data Availability

All data used in this study are fully described in the figure and tables. All partial genomes sequence data of SARS- CoV-2 included in this study are deposited at GenBank (https://www.ncbi.nlm.nih.gov/genbank/) and are available from the corresponding author upon the reasonable request.
